# Perceived risk and associated factors of healthcare waste in selected hospitals of Kathmandu, Nepal

**DOI:** 10.1371/journal.pone.0235982

**Published:** 2020-07-13

**Authors:** Sulata Karki, Surya Raj Niraula, Sabita Karki

**Affiliations:** 1 Department of Public Health and Community Programs, Dhulikhel Hospital Kathmandu University Hospital, Dhulikhel, Nepal; 2 School of Public Health and Community Medicine, B.P. Koirala Institute of Health Sciences, Dharan, Nepal; 3 National Academy of Medical Sciences, Bir Hospital, Kathmandu, Nepal; University of Brescia, ITALY

## Abstract

**Background:**

Healthcare waste management is the subject of a neglected issue in many developing countries. Health care facilities are facing a major challenge in handling healthcare wastes and reducing their potential risks to human health and the environment. Insufficient understanding of the risk associated with healthcare waste by health workforce can contribute to poor waste management practices. The main aim of this study is to assess risk perception towards healthcare waste among hospital attendants and to identify associated factors.

**Methodology:**

We carried out a cross-sectional hospital-based study among 120 attendants of a private and public hospital in Kathmandu, Nepal. We used two-stage random sampling for the selection of hospital and participants. We conducted a face-to-face interview with the participants using semi-structured questionnaires. Based on the mean score, we classified risk perception as good and poor. Bivariate and multivariate analysis was carried out to determine associates of risk perception towards healthcare waste.

**Results:**

Approximately 51.0% of hospital attendants had poor risk perception of healthcare waste. Nearly half of the participants (49.2%) had inadequate knowledge and 43.0% had a negative attitude. Factors such as healthcare waste management training (p = 0.028), housekeeping department (p = 0.036) and attitude (p = 0.001) were associated with risk perception of healthcare waste.

**Conclusion:**

Hospital attendants had a poor understanding of risk perception of healthcare waste. Periodic training on healthcare waste management and edification on the risk associated with healthcare waste is essential to boost awareness among all healthcare workers. Communication on behavioral improvements for appropriate waste management must be prioritized to change the perception of health workers.

## Introduction

Healthcare wastes are generated from different health care facilities which comprised of 85% general wastes i.e. non-hazardous, and rest 15% hazardous waste that may be infectious, toxic or radioactive.[1] Infectious waste have been accountable for public health risk and environmental challenges around the world.[2] Healthcare waste is second most dangerous waste after radiation waste.[3] With the increase in global population and expansion of medical facilities, the amount of waste generation is continuously rising.[4]

In a study of epidemiological literature on the health effects of residence near hazardous waste disposal sites in the United Kingdom, it was found that increases in the risk of adverse health effects such as low birth weight, birth defects, certain forms of cancer have been identified near individual landfill sites and in some multi-site studies.[5] Self-reported disease symptoms like fever, headache, dizziness, itching, burning sensation in eyes, skin rash, cough, and accidental injuries from sharps were identified on human scavengers.[6] Complaints of difficulty breathing due to burning healthcare wastes, foul smell from the hospital, children’s exposure to dumping sites with contaminated syringes have been reported to health professional by the people residing around the hospital.[7]

Every year, it is anticipated that over two million healthcare workers are exposed to percutaneous injuries with infected sharps.[8] There is a high perceived risk of HIV infection caused by healthcare waste in the United States of America.[9] The actual risk of contracting Hepatitis B from the handling of healthcare waste is higher than the risk of infection from other routes.[9]

Health care workers and waste handlers are with the maximum risk of injury or infection due to close contact with infected healthcare wastes.[10] It is noted that the release of highly toxic fumes as a result of open burning and incineration of medical waste can cause adverse health effects and also contribute to global warming.[11] Despite small volume of healthcare waste generated within the hospitals, its disposal and proper management is a complex task and requires additional resources as it can pose a risk of infection to waste handlers.[12] The task of waste management is often assigned to less experienced and unskilled healthcare workers, who perform most activities without proper guidance and insufficient protection in resource constraint settings.[13] Healthcare workers, patients, clients, attendants and waste handlers have been responsible for most of the health problems particularly due to infectious wastes such as bloodborne pathogens, if unmanaged properly.[13]

An increasing number of health facilities in South Asia has led to major challenges in managing healthcare wastes. For instance, uncontrolled burning, reuse of disposable items, unintended accidents from improperly discarded sharps are frequent and lead to health risks.[14] One of the biggest challenges in managing waste can be poor practices as well as substandard information on healthcare waste.[15]

According to the Government of Nepal, a total of 274 hospitals produce 10,520 tons of non-hazardous and 3,094 tons of hazardous medical waste per year. These wastes disposed of with normal municipal garbage was a major concern for waste collectors.[16] A study conducted in some health institutions in different parts of Nepal revealed that 27% of infectious wastes produced from such institutions were mixed with general waste contributing to the infectious waste to 55%.[17] In Nepal, one of the manifested impacts of mismanagement of waste is the alarming incidence of hospital-acquired infection as many institutions are dumping waste on the back yard, rivers, open field, corners of hospital buildings, or anywhere around the premises.[18] Community residents (28%) living around the hospital of Nepal reported complaints of viral fever, cough, cold (difficulty breathing symptoms) and hospital malodor.[19]

Only 38.7% of hospitals have adopted the correct process for segregation of healthcare wastes as per the Ministry of Health and Population, Nepal in 2012.[20] The compliance with healthcare waste management has not been consistent with Nepal’s recommended guidelines for most health care institutions.[21]

Appropriate management of healthcare waste has been a major concern especially in Kathmandu, Nepal.[22] Little attention has been given to healthcare waste management in Nepal as well. There is no precise understanding of the risk associated with healthcare waste especially among waste handlers.[23,24] Hence, evidence on risk perception of healthcare waste among health care workers is needed to ensure better management practices and safety of the health workforce.

## Methods

### Study design and settings

We conducted a cross-sectional study amongst 120 hospital attendants in Kathmandu, Nepal. We collected data between the period of September and November2017 in selected hospitals.

### Sampling technique and sample size

Kathmandu, a densely populated city of Nepal has a large number of hospitals with tertiary treatment facilities.[25] As per the data from the Department of Health Services 2017, a total of 14 hospitals were reported with 150 beds and more.[19] Hospitals holding 150 beds and more, and attendants who consented to participate were inclusion criteria. A two-stage random sampling technique was used to select hospitals and participants. Initially, 1 public hospital and 1 private hospital were randomly selected out of a total of 7 public and 7 private hospitals. The selected public hospital had a total of 164 hospital attendants and the private hospital had 142 attendants. From each of the hospitals, 60 attendants were randomly selected in the second stage. Sample size was calculated as per the study of Muluken Azage et al., which reported 60.0% healthcare workers had adequate risk perception of healthcare waste.[26] Using formula n = z^2^pq/d^2^ where z = 1.96 at 95% confidence interval, prevalence (p) = 60%, compliment of prevalence (q) = 100–60 = 40%, d = 15% of p = 9 and putting all the values, n = 1.96*1.96*60*40/81 = 114 and adding 5% non-response rate, the final sample size was 120.

### Data collection and analysis

We developed a semi-structured questionnaire by reviewing different literature. Face to face interview was conducted with the participants. The questionnaire was finalized in English and then translated into Nepali language using the translation-back translation method. The interview was conducted in the Nepali language. Pretesting of the tool was done by administering the tool to 10% of the total sample in a similar setting. Collected data were entered in Microsoft Excel 2007. The internal consistency was measured via Cronbach’s alpha, which was found to be 0.72 for the Likert scale questions.

Data were processed in the form of tabulation and necessary categorization was done. Microsoft excel was converted into SPSS version 21.0 for statistical analysis. Chi-square test, Fisher’s exact test were used to analyze the association in bivariate analysis. Explanatory variables that were significant in the bivariate analysis at <0.2 were taken for multivariate analysis. Binary logistic regression was performed to estimate odds ratio with 95% confidence interval.

### Ethical approval

Ethical approval was obtained from the Institutional Review Committee, BPKIHS, Dharan (*IRC approval number*: *Acd/783/074/075)*. Aapproval was also obtained from the Ethical Review Committee of respective hospitals (*Ref*: *204 and Ref*: *20112017)*. The respondents were informed about the purpose of data collection. The information was used only for the research purpose. Written informed consent was taken from all the participants. The participants were assured about confidentiality and they had full authority to accept or refuse to take part in the study. The researcher herself collected the data and confidentiality was maintained throughout the study.

## Results

Table 1 depicts the socio-demographic characteristics of 120 respondents with a response rate of 100%. The mean age of hospital attendants was 41.94 with a standard deviation of 8.84 years. Most of the respondents (76.0%) were females. Educational records of respondents displayed, 42.5% had a middle school certificate followed by illiterate (31.7%). The majority (86.7%) of respondents were married and more than half (51.7%) of hospital attendants worked in the inpatient department of the hospital.

**Table 1:** Socio-demographic characteristics of respondents (n = 120).

Characteristics	Categories	Frequency	Percent (%)
**Age (years)**	<25	3	2.5
	25–35	20	16.6
	35–45	53	44.2
	≥45	44	36.7
**Mean age in years ± SD (Min-Max)**		41.94 ± 8.84 (23–59)	
**Sex**	Male	29	24.2
	Female	91	75.8
**Ethnicity**	Disadvantage Janajatis	14	11.7
	Disadvantaged Non-Dalit Terai caste	3	2.5
	Relatively advantaged Janajatis	36	30.0
	Upper caste group	67	55.8
**Education**	Illiterate	38	31.7
	Primary school	28	23.3
	Middle school	51	42.5
	High school	3	2.5
**Marital status**	Unmarried	7	5.8
	Married	104	86.7
	Others	9	7.5
**Religion**	Hindu	110	91.7
	Buddhist	8	6.6
	Christian	2	1.7
**Working department**	Housekeeping	23	19.2
	Inpatient	62	51.6
	Outpatient	24	20.0
	Operation Theatre	3	2.5
	Emergency	3	2.5
	Non clinical	5	4.2
**Work experience (years)**	<10	54	45.0
	10–20	33	27.5
	≥20	33	27.5
**Mean work experience in years ± SD (Min-max)**		13.68 ± 8.21 (1–32)	
**Monthly income (NRs)**	<10000	22	18.3
	10000–20000	92	76.7
	≥20000	6	5.0
**Mean income in rupees ± SD (Min-Max)**		15,703.3± 3,965.4 (9,000–30,000)	

### Knowledge and attitude towards healthcare waste (n = 120)

**Fig 1:**
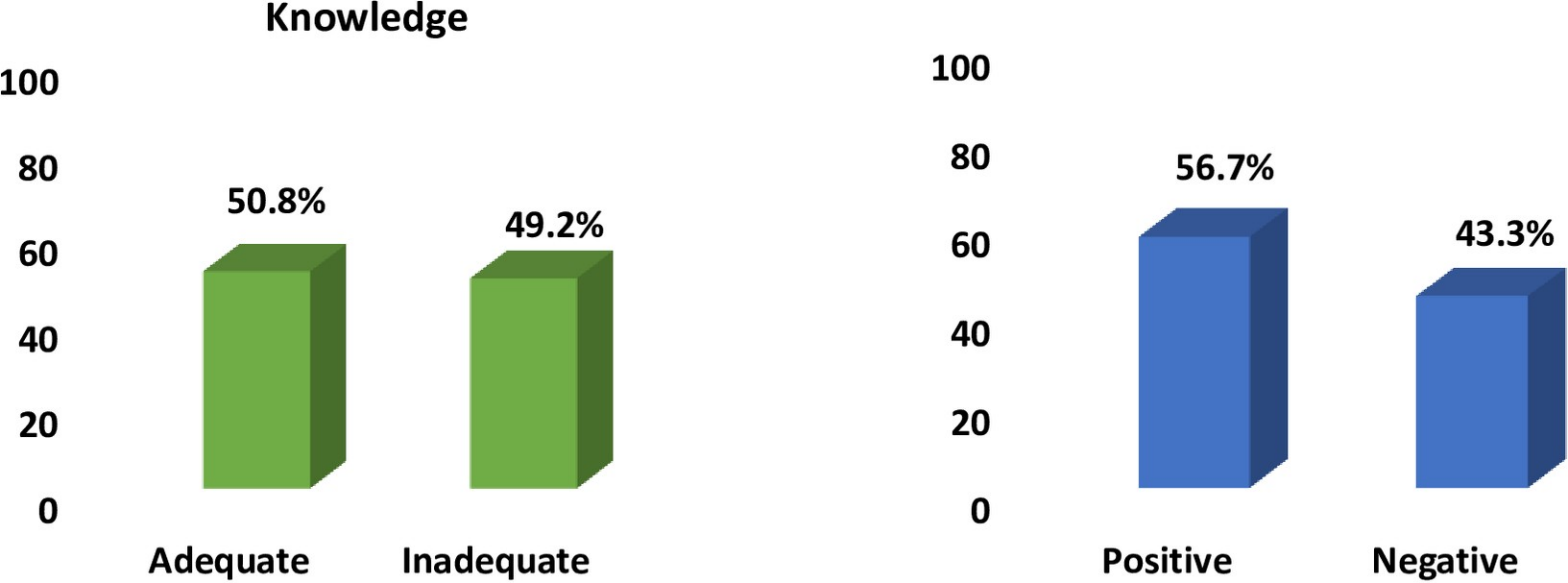
Knowledge and attitude of healthcare waste.

Knowledge was assessed based on 11 questions, classified as adequate and inadequate based on the mean percentage score. Similarly, attitude on 11 statements, classified as positive and negative based on the mean percentage score. Nearly half (49.2%) of the respondents had inadequate knowledge and 43.3% of participants had a negative attitude towards healthcare waste management. (Fig 1)

### Risk perception of healthcare waste

Risk perception was assessed based on 10 statements and later dichotomized as good and poor perception based on mean score. Only 40.0% of respondents agreed that hepatitis B and C may be transmitted through infectious healthcare waste whereas 43.3% agreed that HIV/AIDS may be acquired through contact with infectious healthcare waste. Nearly 52% of the respondents strongly agreed that improperly managed waste may cause infections among health workers and patients. Half of the respondents (50.8%) had poor risk perception while the remaining 49.2% had good risk perception towards healthcare waste. (Table 2)

**Table 2:** Risk perception of hospital attendants towards healthcare waste (n = 120).

Statements	Strongly Agree	Agree	Neutral	Disagree	Strongly Disagree
	n (%)	n (%)	n (%)	n (%)	n (%)
Hepatitis B and C may be transmitted through infectious healthcare waste	39 (32.5)	48 (40.0)	25 (20.8)	8 (6.7)	0 (0.0)
HIV/AIDS may not be acquired through contact with infectious healthcare waste*	10 (8.3)	29 (24.2)	10 (8.3)	52 (43.3)	19 (15.9)
Improperly managed waste may cause infections among health workers and patients	62 (51.7)	58 (48.3)	0 (0.0)	0 (0.0)	0 (0.0)
Improperly managed healthcare waste may cause cancer in future	27 (22.5)	61 (50.8)	23 (19.2)	7 (5.8)	2 (1.7)
Sharp waste like needles, blades, broken glasses are harmful to human health	86 (71.7)	34 (28.3)	0 (0.0)	0 (0.0)	0 (0.0)
Proper management of sharps reduces needle stick injury	49 (40.8)	56 (46.7)	7 (5.8)	8 (6.7)	0 (0.0)
Radioactive waste are not harmful to human health*	2 (1.7)	18 (15.0)	27 (22.5)	32 (26.6)	41 (34.2)
Improperly managed waste may contaminate water source	44 (36.8)	67 (55.8)	4 (3.3)	4 (3.3)	1 (0.8)
Proper waste management reduces air pollution	43 (35.8)	64 (53.3)	11 (9.2)	2 (1.7)	0 (0.0)
All healthcare wastes may transmit diseases*	36 (30.0)	43 (35.8)	8 (6.7)	25 (20.8)	8 (6.7)

*Reverse statement

### Associated factors of risk perception

The significant difference with the variables like waste management training, working department and attitude in binary logistic regression analysis. The housekeeping department was less likely to have good risk perception towards healthcare waste compared to other departments. Adjusted Odds Ratio (AOR) = 0.15, 95% Confidence Interval (CI): 0.025–0.886; p = 0.036) Healthcare waste management training was significantly associated with risk perception towards healthcare waste. The odds of perceived risk towards healthcare waste were 3.1 times higher among those who attended training than those who didn’t attend the training. (AOR = 3.12, 95% CI: 1.131–8.621; p = 0.028). The attitude of hospital attendants was another strong predictor for risk perception towards healthcare waste. Attendants who had positive attitude towards healthcare waste perceived risk 4.4 times higher than those who had a negative attitude. (AOR = 4.48; 95% CI: 1.895–10.597; p = 0.001). (Table 3)

**Table 3:** Binary logistic regression for the factors associated with risk perception.

Variables	Categories	β	Odds ratio	95% CI for odds ratio		p value
				Lower	Upper	
Education	Literate	0.702	2.018	0.808	5.038	0.133
	Illiterate	Ref				
Working department	Housekeeping	-1.900	0.150	0.025	0.886	0.036*
	Inpatient	-1.031	0.357	0.075	1.704	0.196
	Outpatient	-1.410	0.244	0.042	1.418	0.116
	Others	Ref				
Monthly income	>10000 NRs	0.106	1.111	0.322	3.834	0.867
	≤10000 NRs	Ref				
Waste management training	Yes	1.139	3.123	1.131	8.621	0.028*
	No	Ref				
Knowledge on HCWM	Adequate	-0.104	0.901	0.353	2.301	0.828
	Inadequate	Ref				
Attitude	Positive	1.500	4.481	1.895	10.597	0.001*
	Negative	Ref				
**Constant**		-0.906	0.404			0.338

*Significant at p<0.05 Ref: Reference

## Discussion

This study primarily aimed to assess the risk perception of hospital attendants towards healthcare waste. It has also assessed knowledge, attitude towards healthcare waste management and identified the factors associated with risk perception. This study showed nearly half (49.2%) of respondents had inadequate knowledge regarding healthcare waste management. The present study is similar to the study done in Cairo where a significant difference was noticed in the knowledge score of physicians, nurses and housekeepers. The knowledge score of physicians (68.3%) was significantly higher than that of nurses (60.9%) and housekeepers (40.4%).[25] A slight difference was noticed in the study done in Bangladesh where nearly two-thirds of technologists and cleaning staff had inadequate knowledge.[27]

Around half (50.8%) of hospital attendants agreed that segregation of waste at source decreases the risk of injury. Nearly two-fifth (36.7%) agreed that decontamination or disinfection decreases the chances of infection and 47.5% agreed with the statement, vaccination against hepatitis B prevents transmission of hospital-acquired infection in this study. The result is consistent with the study done in India where 53.6% of attendees agreed that segregation of waste at source decreases the risk of injury to waste handlers. However, 33.5% of dentists agreed with the same statement.[28] The difference might be due to the responsibility of attendants who are directly involved in handling healthcare waste compared to dentists. Overall, attitude in this study was scored and dichotomized where more than half (56.7%) of respondents had a positive attitude and 43.3% had a negative attitude towards healthcare waste management. Regarding the attitude of healthcare workers towards waste disposal in Cairo it was found that, more housekeeping staff had satisfactory attitude scores (61.9%) than nurses (49.0%) and physicians (56.4%) which is consistent with present study.[25] Study done in Ethiopia revealed that knowledge, attitude, and practices, 25 (45.5%), 43 (78.2%), and 44 (80%) of the study participants had adequate knowledge, favorable attitude, and adequate practice scores respectively.[29]

Different studies in various settings reported that risk perception was different across health professionals. In the present study, more than half (50.8%) of hospital attendants had poor risk perception whereas, 49.2% had good risk perception towards healthcare waste. In the study done in Portugal, nurses were the group with the highest risk perception to waste management operations, except for transport where the highest risk perception was by the housekeepers.[24] A study done in Ethiopia showed medical doctors had better risk perception than other health workers.[26] The difference might be due to educational background, training, knowledge on waste management guidelines, and commitment of healthcare staff. A study in Tanzanian hospitals had concluded with findings that administrators perceived lower rates than implementers.[30] Individuals from urban areas have about 3 times higher odds of increased perceived risk than from rural areas. On the other hand, people living around the hospitals have 2.5 times higher odds of increased perceived risk than those people living near to health centers in a study done among community residents living around the hospital.[31]

In this study, around 42.0% of the hospital attendants were not trained in healthcare waste management. A nearly similar result was seen in a study done in Ethiopia where 53.1% of healthcare workers didn’t take any training on healthcare waste management.[26] The risk perception of respondents towards healthcare waste was significantly associated with waste management training, working department and attitude in this study. The odds of perceived risk towards healthcare waste were 3.1 times higher among those who attend training than those who didn’t attend the training. This supports the findings of Ethiopia, where training on healthcare waste, knowledge on healthcare waste types, and diseases transmitted with healthcare waste was significantly associated with risk perception towards healthcare waste. Besides, the odds of adequate risk perception among healthcare workers who took training were 1.87 times higher than the odds of risk perception among those who didn’t take training on healthcare waste.[26] Similar factor like waste management training was associated in a study done by Wafula ST et al.[32] Knowledge was significantly associated with risk perception towards healthcare waste among community people residing around the hospitals in a study carried out in Kathmandu, Nepal.[19]

## Conclusion

Perceived risk of healthcare waste among hospital attendants was found to be poor as it was surprising to mention half of the attendants were unfamiliar with the risk related to healthcare waste. Additionally, with inadequate knowledge of healthcare waste management. Waste management training, working department and attitude were the significant factors associated with risk perception towards healthcare waste. Periodic training on healthcare waste management and edification on the risk associated with healthcare waste is fundamental to boost awareness among all healthcare workers. Rigorous implementation of healthcare waste management guidelines to ensure proper waste management along with the provision of personal protective equipment for waste handlers helps to reduce injury.

### Limitations

This study was conducted in tertiary hospitals of Nepal with relatively less sample size. Thus, the results might however reduce the maximum credibility, especially while generalizing to whole health care workers. The perception could have been better assessed if a qualitative study was done. Nevertheless, this study might provide evidence to reconsider waste management issues in the context of developing countries.

## Supporting information

S1 Data (XLSX)

## Abbreviations

AOR: Adjusted Odds Ratio
CI: Confidence Interval
HCWM: Healthcare Waste Management
SPSS: Statistical Package for Social Sciences
